# Supplementary material for: An oxylipin signal confers protection against antifungal echinocandins in pathogenic aspergilli
Source: Nat Commun. 2024 May 4;15:3770. doi: 10.1038/s41467-024-48231-2 (PMC11069582; doi:10.1038/s41467-024-48231-2)
Supplement: Supplementary file 1 — Supplementary Information [file 41467_2024_48231_MOESM1_ESM.pdf]

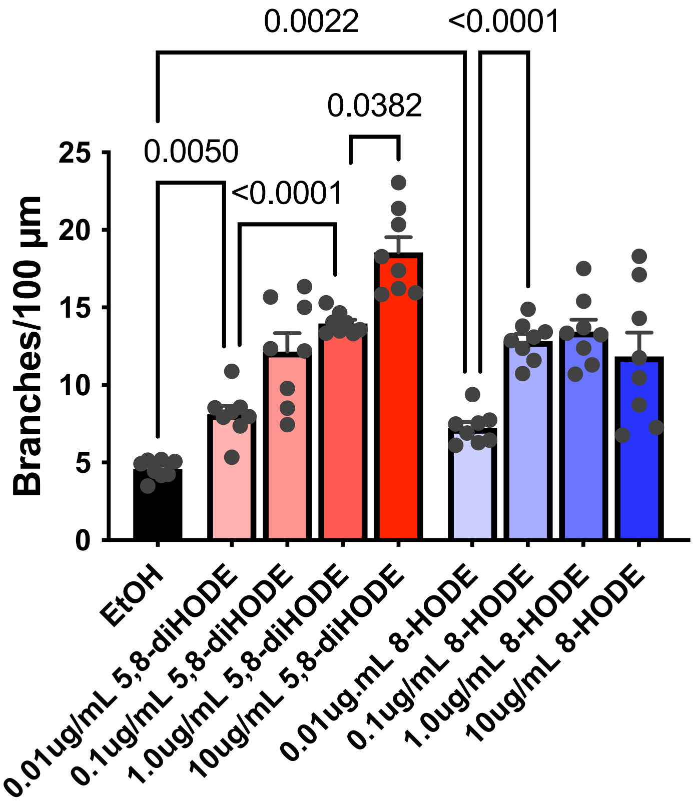

Supplementary Figure 1. 5,8-diHODE and 8-HODE increase lateral branching in WT *A. fumigatus* Af293. Lateral branches per 100 µm of hypha were assessed for eight hyphae of *A. fumigatus* WT Af293 grown for twenty hours in GMM with 1% EtOH, 5,8-diHODE, or 8-HODE treatment. Data points represent individual hyphae (n = 8) and error bars represent SEM. P values were determined by Browne-Forsythe and Welch ANOVA with Dunnett's T3 multiple comparisons tests. * denotes p<0.05, ** denotes p<0.01, and **** denotes p<0.0001.

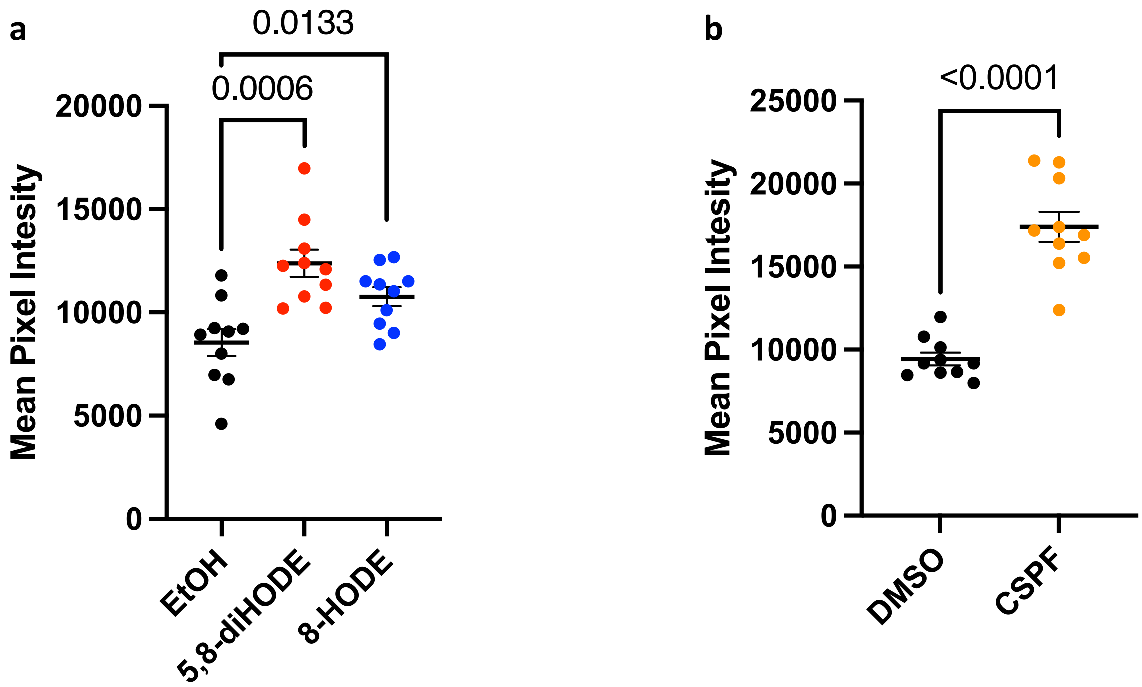

Supplementary Figure 2. 5,8-diHODE, 8-HODE, and caspofungin treatments increase cell wall chitin in *A. fumigatus* hyphae. (a) Mean calcofluor white intensity per pixel of WT Af293 hyphae grown for 15 hours in GMM with 1% EtOH, 1 µg/mL 5,8-diHODE, or 1 µg/mL 8-HODE before staining and epifluorescent imaging. (b) Mean calcofluor white intensity per pixel of WT Af293 hyphae grown for 15 hours in GMM with 1% DMSO or 1 µg/mL caspofungin for 15 hours before staining and epifluorescent imaging. (a,b) Data points represent individual hyphae (n = 10) and error bars represent SEM. P values shown were calculated using two-sided Welch's t-test.

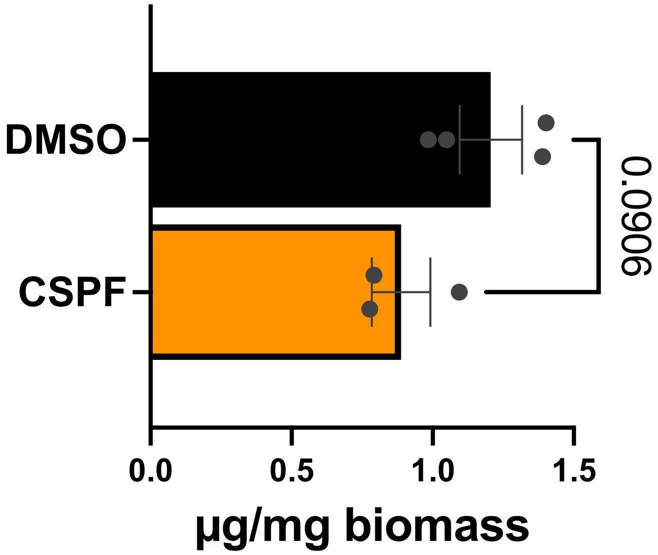

Supplementary Figure 3. Caspofungin treatment does not impact 10-HODE production by PpoC. 10-HODE per milligram of dry biomass extracted from fungal tissue. WT *A. fumigatus* Af293 was grown at 37˚ C and 250 RPM for 24 hours in GMM plus 48 hours more after the addition of 0.02% DMSO (n = 4) or 2 µg/mL CSPF (n = 3). Oxylipins were extracted using mixed organic solvent and quantified on UHPLC-MS/MS by comparison to standard curves of purified oxylipin. Data points represent independent culture flasks and error bars denote SEM. P value shown was determined by Welch’s two-sided t-test.

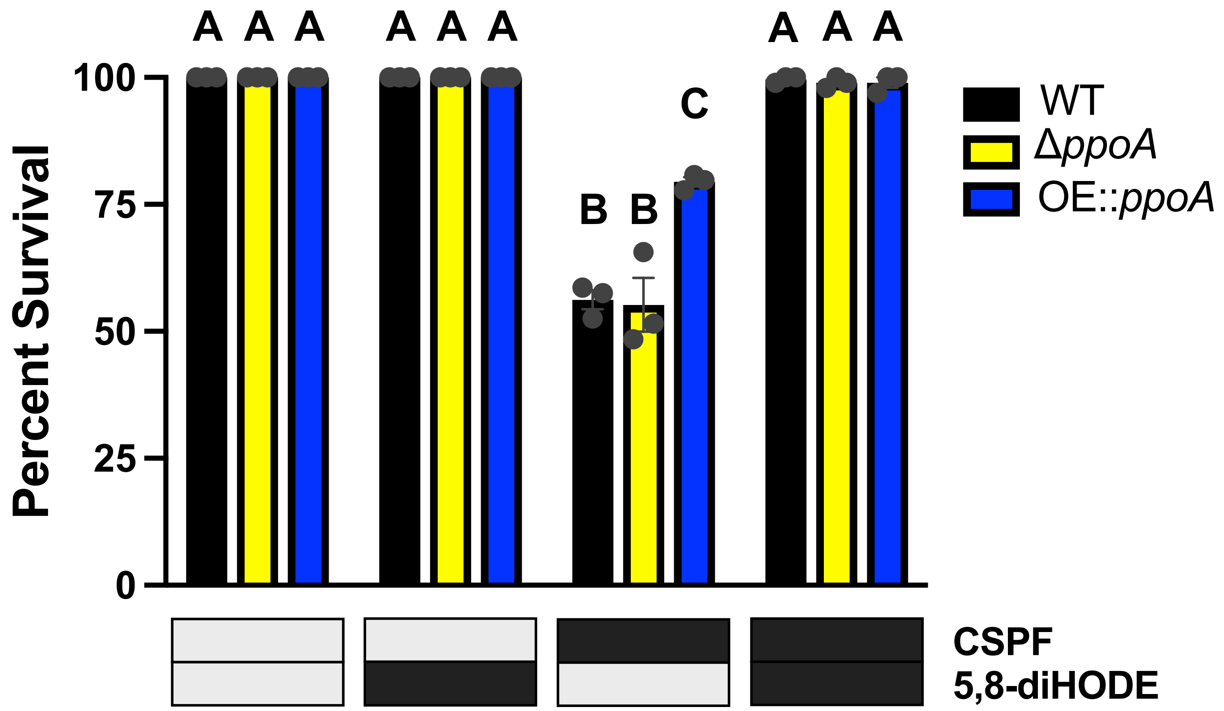

Supplementary Figure 4. Overexpression of *ppoA* is protective against echinocandin tip lysis. Percent survival of *A. fumigatus* Af293 WT, Δ*ppoA*, and OE::*ppoA* germlings treated with 1 µg/mL CSPF or 1% DMSO vehicle and 10 µg/mL 5,8-diHODE or 1% EtOH vehicle after 16 hours at 37˚ C in YMM. Data points each represent percent survival of 99 germlings assessed in biologically independent samples (n = 3) with errors bars representing SEM. Conditions with p values less than 0.05 calculated by two-way ANOVA with Tukey's multiple comparisons are indicated by distinct letters. Exact p values are provided in the source data file.

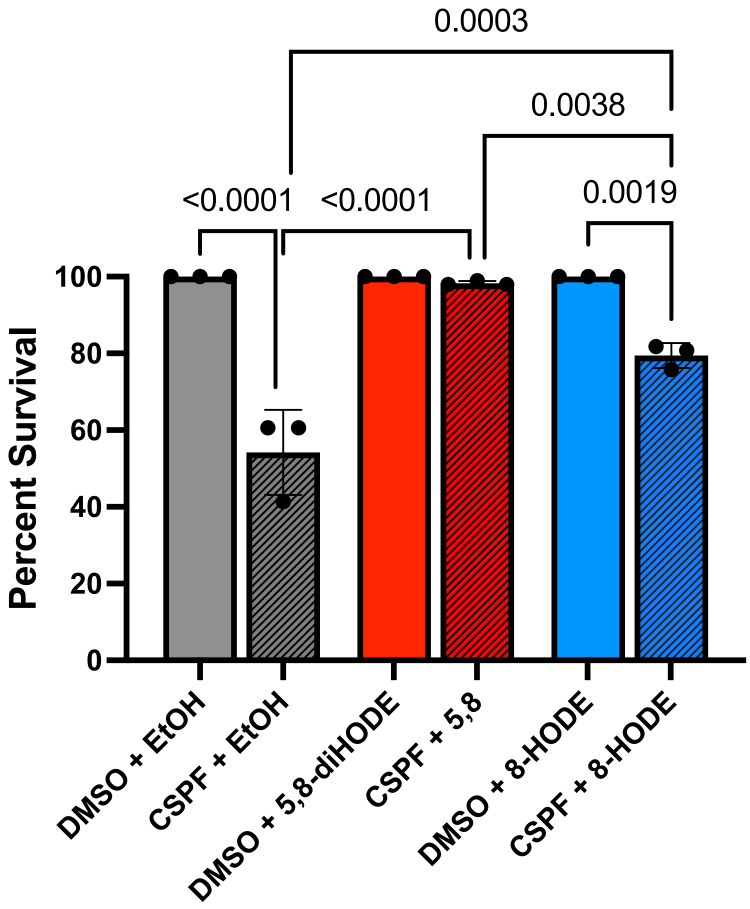

Supplementary Figure 5. Cotreatment with 5,8-diHODE protects WT *A. fumigatus* AfS35 against caspofungin mediated tip lysis. Percent survival of WT *A. fumigatus* AfS35 germlings treated with 2 µg/mL CSPF or 1% DMSO vehicle and 10 µg/mL 5,8-diHODE, 10µg/mL 8-HODE or 1% EtOH vehicle after 16 hours at 37˚ C in GMM. P values shown were calculated by one-way ANOVA with Tukey's multiple comparisons. Data points each represent percent survival of 99 germlings assessed in biologically independent samples (n = 3) with errors bars representing SEM.

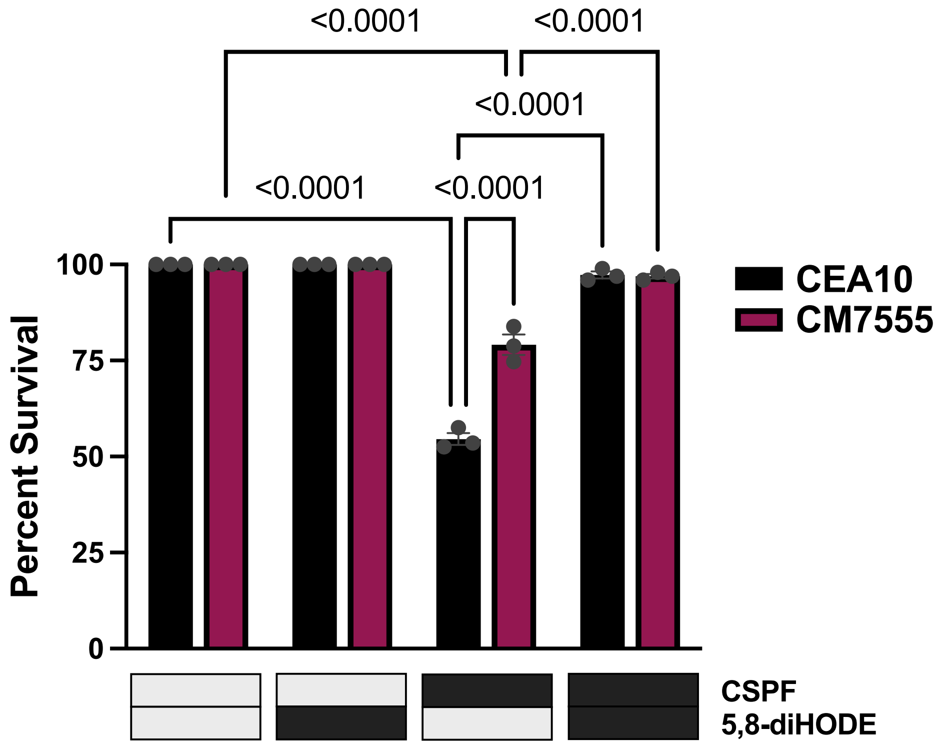

Supplementary Figure 6. Cotreatment with 5,8-diHODE protects WT *A. fumigatus* CM7555 against caspofungin mediated tip lysis. Percent survival of WT *A. fumigatus* isolates CEA10 and CM7555 germlings treated with 2 µg/mL CSPF or 1% DMSO vehicle and 10 µg/mL 5,8-diHODE or 1% EtOH vehicle after 16 hours growth in GMM. P values were calculated by two-way ANOVA with Tukey's multiple comparisons. Data points each represent percent survival of 99 germlings assessed in biologically independent samples (n = 3) with errors bars representing SEM.

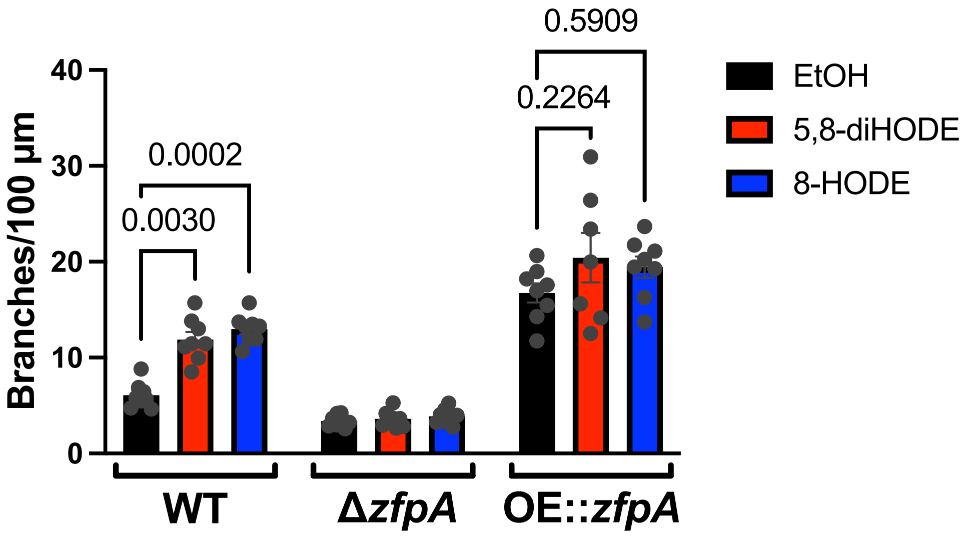

Supplementary Figure 7. ZfpA is necessary for hyperbranching response to the PpoA oxylipins 5,8-diHODE and 8-HODE. Lateral branches per 100 µm of hypha were assessed for seven or eight hyphae of *A. fumigatus* Af293 WT, Δ*zfpA*, and OE::*zfpA* strains grown for twenty hours in GMM with 1% EtOH, 0.1 µg/mL 5,8-diHODE, or 0.1 µg/mL 8-HODE. Data points represent individual hyphae (n = 7 or 8) and error bars represent SEM. P values shown were determined by two-way ANOVA with Tukey's multiple comparisons tests.

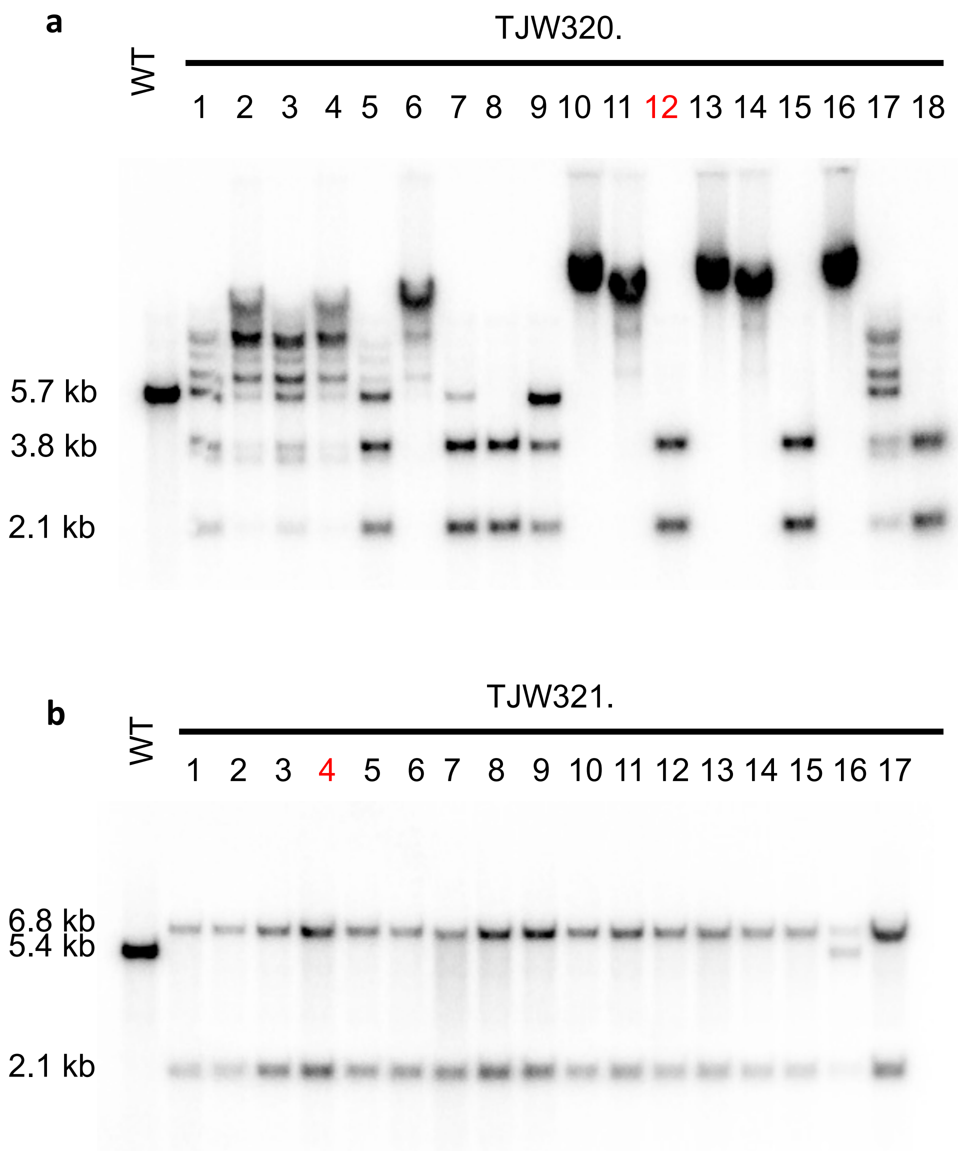

Supplementary Figure 8. Southern blot confirmation of *A. flavus zfpA* mutants. (A) Deletion mutant Southern confirmation. Genomic DNA was digested by *EcoR*I. Wild type band should be present at 5.7 kb and Δ*zfpA* bands at 3.8 and 2.1 kb. TJW320.12 was chosen for the subsequent experiments. (B) Overexpression mutant Southern confirmation. Genomic DNA was digested by *Nco*I. Wild type band should be present at 5.4 kb and OE::*zfpA* bands at 6.8 and 2.1 kb. TJW321.4 was chosen for the subsequent experiments.

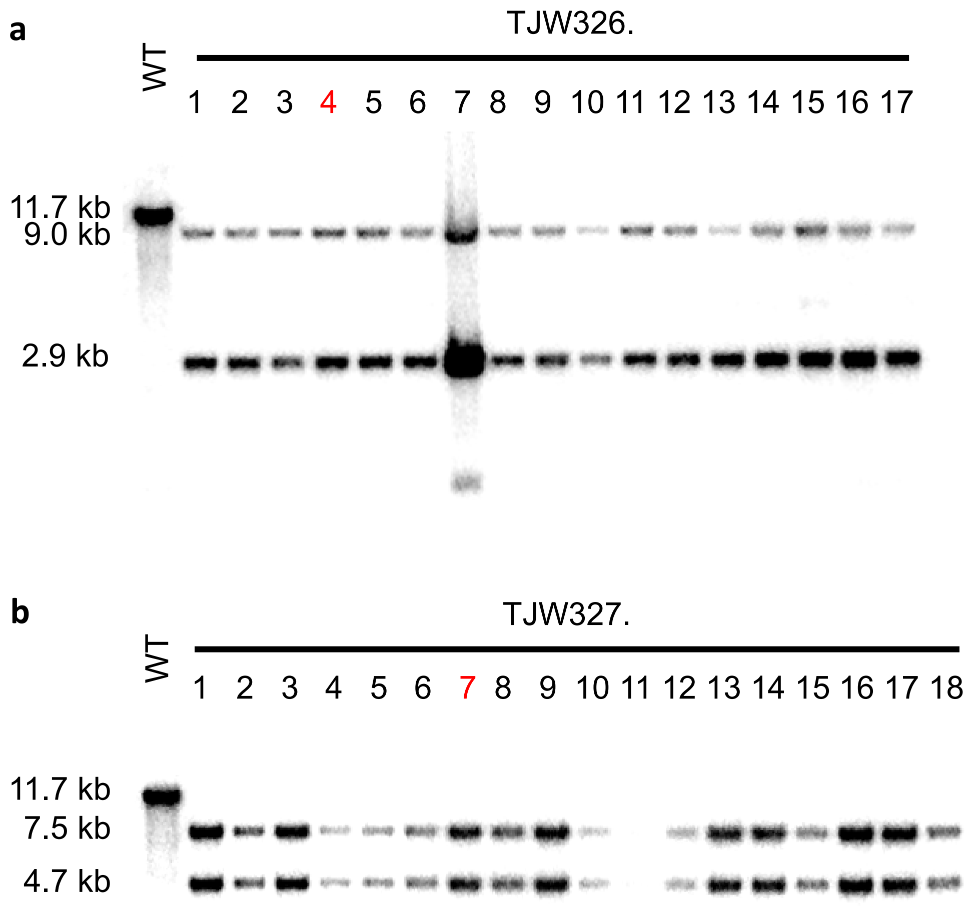

Supplementary Figure 9. Southern blot confirmation of *A. nidulans zfpA* mutants. (A) Deletion mutant Southern blot confirmation. Genomic DNA was digested by *Pvu*II. Wild type band can be observed at 11.7 kb and Δ*zfpA* bands at 9 and 2.9 kb. TJW326.4 was chosen for the subsequent experiments. (B) Overexpression mutant Southern confirmation. Genomic DNA was digested by *Pvu*II. Wild type band can be observed at 11.7 kb and OE::*zfpA* bands at 7.5 and 4.7 kb. TJW327.7 was chosen for the subsequent experiments.

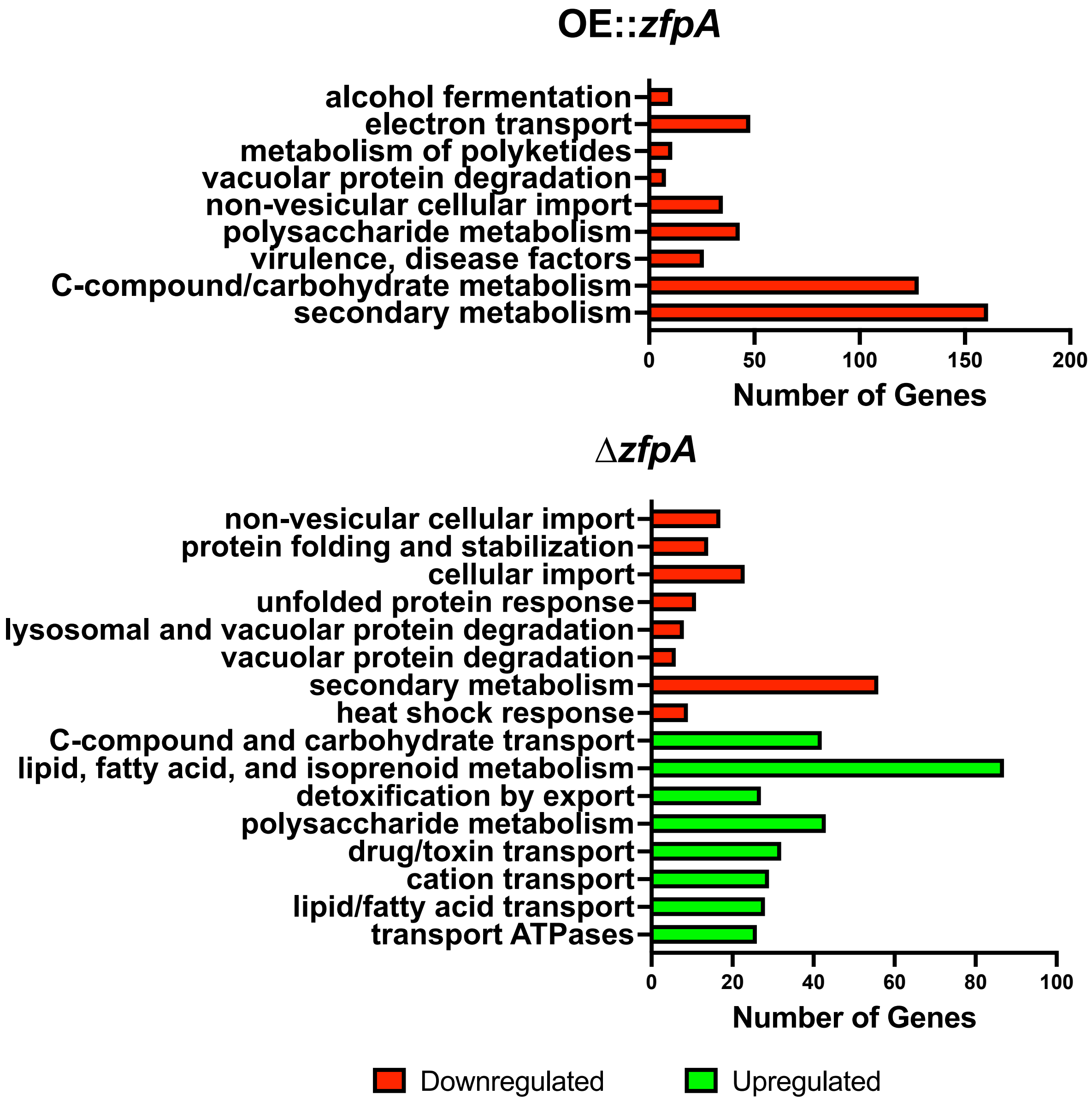

Supplementary Figure 10. ZfpA regulates genes involved in diverse cellular processes. Functional category enrichment of genes significantly up- and downregulated in the OE::*zfpA* and Δ*zfpA* mutants relative to WT Af293 (Benjamini-Hochberg FDR < 0.05). Differentially expressed genes were defined as |Log2FC| ≥ 1 and p. adj. < 0.01.

**Strain Name**	**Background**	**Organism**	**Genotype**	**Source**
WT Af293	Af293	*A. fumigatus*	Wild type	Clinical Isolate
TDWC1.13	Af293	*A. fumigatus*	*pyrG1*; Δ*ppoA*::*A.p.pyrG*	[1]
WT CEA10	CEA10	*A. fumigatus*	Wild type	Clinical Isolate
CM7555	CM7555	*A. fumigatus*	Wild type	[2]
AfS35	D141	*A. fumigatus*	Δ*akuA::loxP*	[3]
TFYL81.5	Af293	*A. fumigatus*	*pyrG1*; *argB1*; Δ*akuA*::mluc; *A.fu.argB*; *A.fu.pyrG*	[4]
TJW213.1	Af293	*A. fumigatus*	*pyrG1*; *argB1*; Δ*akuA*::mluc; *A.fu.argB*; Δ*zfpA*::*A.p.pyrG*	[5]
TJW214.2	Af293	*A. fumigatus*	*pyrG1*; *argB1*; Δ*akuA*::mluc; *A.fu.argB*; *A.p.pyrG*:: *A.n.gpdA (p)*::*zfpA*	[5]
TDGC1.2	CEA10	*A. fumigatus*	*pyrG1*; Δ*akuB*; Δ*argB*; *A.fu.argB::A.n.gpdA(p)*::RFP; *A.fu.pyrG*	[5]
TJW215.1	CEA10	*A. fumigatus*	*pyrG1*; Δ*akuB*; Δ*argB*; *A.fu.argB::A.n.gpdA(p)*::RFP; Δ*zfpA*::*A.p.pyrG*	[5]
TJW216.1	CEA10	*A. fumigatus*	*pyrG1*; Δ*akuB*; Δ*argB*; *A.fu.argB::A.n.gpdA(p)*::RFP; *A.p.pyrG*:: *A.n.gpdA(p)*::*zfpA*	[5]
ΔakuB^{KU80}	CEA10	*A. fumigatus*	Δ*akuB*::*A.fu.pyrG*	[6]
Δ*mpkA*	CEA10	*A. fumigatus*	Δ*akuB*::*pyrG*; *mpkA*::*ptrA*	[7]
mpkA^{C}	CEA10	*A. fumigatus*	Δ*akuB*::*pyrG*; *mpkA*::*mpkA*$^{+}$::*hygR*	[7]
Δcrz*A*	CEA10	*A. fumigatus*	Δ*akuB*; Δ*crzA*::*pyrG*	[8]
crzA^{C}	CEA10	*A. fumigatus*	Δ*akuB*; Δ*crzA*::*crzA*$^{+}$::*pyrG*	[8]
WT NRRL 3357	NRRL 3357	*A. flavus*	Wild type	Peanut Isolate
WT FGSC A4	FGSC A4	*A. nidulans*	Wild type	Soil Isolate
TJW149.27	NRRL 3357	*A. flavus*	Δ*nkuA*	[9]
TJES19.1	NRRL 3357	*A. flavus*	*pyrG-*; Δ*nkuA*	[10]
TJ320.12	NRRL 3357	*A. flavus*	*A.p.pyrG::A.n.gpd(p)::Afl.zfpA;* Δ*ku70*	This study
TJW321.4	NRRL 3357	*A. flavus*	*A.p.pyrG::A.n.gpd(p)::A.fl.zfpA;* Δ*ku70*	This study
RDIT9.32	FGSC A4	*A. nidulans*	*veA*	[11]
RJMP1.49	FGSC A4	*A. nidulans*	*pyrG89*; *veA*	[12]
RTMH217.13	FGSC A4	*A. nidulans*	*pyrG89*; *veA*	[13]

TJW326.4	FGSC A4	*A. nidulans*	Δ*A.n.zfpA*::A.p.*pyrG; pyroA4,* Δn*kuA::argB; veA*	This study
RJW343.2	FGSC A4	*A. nidulans*	Δ*A.n.zfpA*::A.p.*pyrG; veA*	This study
TJW327.7	FGSC A4	*A. nidulans*	*A.p.pyrG::A.n.gpd(p)::A.n.zfpA,* pyroA4; Δn*kuA::argB; veA*	This study
RJW344.1	FGSC A4	*A. nidulans*	*A.p.pyrG::A.n.gpd(p)::A.n.zfpA, veA*	This study

Supplementary Table 1. Fungal strains used in this study.

Name	5' → 3'
***A. nidulans zfpA* knock out**	
Anzfpko5'F	TCATCAGCAGCATCATCGTCGG
Anzfpko5'R	CGATATCAAGCTATCGATACCTCGACTCTTT<br>GACGGTGTTGGATCGCGATGAGAGTCG
parapyrGF	GTCGACGGTATCGATAAGCTTG
parapyrGR	ATTCGACAATCGGAGAGGCTGC
Anzfpko3'F	GTCGCTGCAGCCTCTCCGATTGTCGAA<br>TCTTATATGCATGATGATAGCGGCATTTTGG
Anzfpko3'R	TGCAATGACATGTCCCTCACCC
AnzfpkoconfF	TAAGCCTAGTTCCTCACACGCC
AnzfpkoconfR	TAGGGCCTATCCTTAGGGTACC
***A. nidulans zfpA* overexpression**	
AnzfpOE5'F	TCATCAGCAGCATCATCGTCGG
AnzfpOE5'R	CCAATTCGCCCTATAGTGAGTCGTATTA<br>CGTTTGACGGTGTTGGATCGCGATGAGAG
OEPyGF	CGTAATACGACTCACTATAGGGC
OEPyGR	GGTGATGTCTGCTCAAGCGGG
AnzfpOE3'F	CAGCTACCCCGCTTGAGCAGACATCACCAT<br>GATGGCACTAGAGCCTTCAAGGCAAC
AnzfpOE3'R	AAGCCCTTGATGTGGTACTCGC
AnzfpOEconfF	ATTCATCTTCCCATCCAAGAACC
AnzfpOEconfR	ATCTTGGCAGTAGTCCGAGACG
***A. flavus zfpA* knock out**	
Aflzfpko5'F2	TCTGGCGCTCCACATTTCAACC
Aflzfpko5'R	CGATATCAAGCTATCGATACCTCGACT<br>CCAAGGGTTGGTTATTTTCAGTATGTGGAG
ParapyrGF	GAGTCGAGGTATCGATAAGCTTG
ParapyrGR	ATTCGACAATCGGAGAGGCTGC
Aflzfpko3'F	GTCGCTGCAGCCTCTCCGATTGTCGAA<br>TGAGACTCCAGTTTCGCAAGTTCGGGTC
Aflzfpko3'R	ACTACATCGATCAAGGGCTGCC
AflzfpkoconfF	AGCGGAAAGAGGTCAACACAGG
AflzfpkoconfR	TAGGGCCTATCCTTAGGGTACC
***A. flavus zfpA* overexpression**	
AflzfpOE5'F	TCTGGCGCTCCACATTTCAACC
AflzfpOE5'R	CCAATTCGCCCTATAGTGAGTCGTATTACGC<br>AAGGGTTGGTTATTTTCAGTATGTGGAG
OEPyGF	CGTAATACGACTCACTATAGGGC
OEPyGR	GGTGATGTCTGCTCAAGCGGG

AflzfpOE3'F	CAGCTACCCCGCTTGAGCAGACATCACC ATGCAAGGCCACAGTGACCATCCTGACTTTG
AflzfpOE3'R	AAAAGCCGCAGACCATTGTGCC
AflzfpOEconfF	ATTCATCTTCCCATCCAAGAACC
AflzfpOEconfR	AGTCAAGTGGCGCTTGAAGACG
**Probe templates for Northerns**	
ppoA_NorthernF	ACAGGACCTCATCAGGACGTTC
ppoA_NorthernR	AAGTTGGTGCAGAGTCCACTGC
A.fl.ppoA_NorthernF	TCATATCTCGATCTCTCGCCGC
A.fl.ppoA_NorthernR	AGGGTAGATCTCCACATGGTCC
A.fl.gpdA_NorthernF	CTCAAATACGACACCACCCACG
A.fl.gpdA_NorthernR	TCCTCGGAAGCAGCCTTGATGG
A.n.ppoA_NorthernF	TTCGCTACCAAGCGAGTGTTGG
A.n.ppoA_NorthernR	TCTATCACCGCGAACCAATGCC
A.n.gpdA_NorthernF	GTATGACTCACAGCACGGTCAG
A.n.gpdA_NorthernR	CTTGAGCTCGTTCTCAGAAGCC

Supplementary Table 2. Primers used in this study.

Supplementary References

1. Dagenais, T. R. T. *et al.* Defects in conidiophore development and conidium-macrophage interactions in a dioxygenase mutant of *Aspergillus fumigatus*. *Infect. Immun.* **76**, 3214–3220 (2008).

2. Valero, C. *et al.* The Caspofungin Paradoxical Effect is a Tolerant "Eagle Effect" in the Filamentous Fungal Pathogen *Aspergillus fumigatus*. *MBio* **13**, e0044722 (2022).

3. Wagener, J. *et al.* The putative alpha-1,2-mannosyltransferase AfMnt1 of the opportunistic fungal pathogen *Aspergillus fumigatus* is required for cell wall stability and full virulence. *Eukaryotic Cell* **7**, 1661–1673 (2008).

4. Throckmorton, K., Lim, F. Y., Kontoyiannis, D. P., Zheng, W. & Keller, N. P. Redundant synthesis of a conidial polyketide by two distinct secondary metabolite clusters in *Aspergillus fumigatus*. *Environ. Microbiol.* **18**, 246–259 (2016).

5. Schoen, T. J. *et al.* *Aspergillus fumigatus* transcription factor ZfpA regulates hyphal development and alters susceptibility to antifungals and neutrophil killing during infection. *PLoS Pathog.* **19**, e1011152 (2023).

6. da Silva Ferreira, M. E. *et al.* The akuB(KU80) mutant deficient for nonhomologous end joining is a powerful tool for analyzing pathogenicity in *Aspergillus fumigatus*. *Eukaryotic Cell* **5**, 207–211 (2006).

7. Valiante, V., Jain, R., Heinekamp, T. & Brakhage, A. A. The MpkA MAP kinase module regulates cell wall integrity signaling and pyomelanin formation in *Aspergillus fumigatus*. *Fungal Genet. Biol.* **46**, 909–918 (2009).

8. Soriani, F. M. *et al.* Functional characterization of the *Aspergillus fumigatus* CRZ1 homologue, CrzA. *Mol. Microbiol.* **67**, 1274–1291 (2008).

9. Zhao, X. *et al.* A Cellular Fusion Cascade Regulated by LaeA Is Required for Sclerotial Development in *Aspergillus flavus*. *Front. Microbiol.* **8**, 1925 (2017).

10. Frawley, D. *et al.* The tetrameric pheromone module SteC-MkkB-MpkB-SteD regulates asexual sporulation, sclerotia formation and aflatoxin production in *Aspergillus flavus*. *Cell. Microbiol.* **22**, e13192 (2020).

11. Tsitsigiannis, D. I., Kowieski, T. M., Zarnowski, R. & Keller, N. P. Endogenous lipogenic regulators of spore balance in *Aspergillus nidulans*. *Eukaryotic Cell* **3**, 1398–1411 (2004).

12. Shaaban, M. *et al.* Involvement of transposon-like elements in penicillin gene cluster regulation. *Fungal Genet. Biol.* **47**, 423–432 (2010).

13. Hammond, T. M., Andrewski, M. D., Roossinck, M. J. & Keller, N. P. *Aspergillus* mycoviruses are targets and suppressors of RNA silencing. *Eukaryotic Cell* **7**, 350–357 (2008).
