# Supplementary material for: An oxylipin signal confers protection against antifungal echinocandins in pathogenic aspergilli
Source: Nat Commun. 2024 May 4;15:3770. doi: 10.1038/s41467-024-48231-2 (PMC11069582; doi:10.1038/s41467-024-48231-2)
Supplement: Supplementary file 3 — Description of Additional Supplementary Files [file 41467_2024_48231_MOESM3_ESM.pdf]

Description of Additional Supplementary Files:

Supplementary Data 1: Differentially expressed genes (DEGs) identified in the RNA sequencing experiment. All DEGs with an adjusted p value < 0.01 are listed with Log_2FC, P-values, and adjusted P values designated for each gene.

Supplementary Movie 1: Time-lapse of *A. fumigatu*s Af293 WT conidia treated with 1% DMSO plus 1% EtOH grown for 24 hours in liquid YMM at 37˚C. Images were collected every 15 minutes for 21 hours beginning at 3 hours post inoculation by phase microscopy with a 10X objective. Scale bars represent one-hundred microns.

Supplementary Movie 2: Time-lapse of *A. fumigatus* Af293 WT conidia treated with 1% DMSO plus 10 µg/mL 5,8-diHODE grown for 24 hours in liquid YMM at 37˚C. Images were collected every 15 minutes for 21 hours beginning at 3 hours post inoculation by phase microscopy with a 10X objective. Scale bars represent one-hundred microns.

Supplementary Movie 3: Time-lapse of *A. fumigatus* Af293 WT conidia treated with 1 µg/mL caspofungin plus 1% EtOH grown for 24 hours in liquid YMM at 37˚C. Images were collected every 15 minutes for 21 hours beginning at 3 hours post inoculation by phase microscopy with a 10X objective. Scale bars represent one-hundred microns.

Supplementary Movie 4: Time-lapse of *A. fumigatus* Af293 WT conidia treated with 1 µg/mL caspofungin plus 10 µg/mL 5,8- diHODE grown for 24 hours in liquid YMM at 37˚C. Images were collected every 15 minutes for 21 hours beginning at 3 hours post inoculation by phase microscopy with a 10X objective. Scale bars represent onehundred microns.

Supplementary Movie 5: Time-lapse of *A. fumigatus* CEA10 WT conidia treated with 1% DMSO plus 1% EtOH grown for 24 hours in liquid GMM at 37˚C. Images were collected every 15 minutes for 21 hours beginning at 3 hours post inoculation by phase microscopy with a 10X objective. Scale bars represent one-hundred microns.

Supplementary Movie 6: Time-lapse of *A. fumigatus* CEA10 WT conidia treated with 1% DMSO plus 10 µg/mL 5,8-diHODE grown for 24 hours in liquid GMM at 37˚C. Images were collected every 15 minutes for 21 hours beginning at 3 hours post inoculation by phase microscopy with a 10X objective. Scale bars represent one-hundred microns.

Supplementary Movie 7: Time-lapse of *A. fumigatus* CEA10 WT conidia treated with 2 µg/mL caspofungin plus 1% EtOH grown for 24 hours in liquid GMM at 37˚C. Images were collected every 15

minutes for 21 hours beginning at 3 hours post inoculation by phase microscopy with a 10X objective. Scale bars represent one-hundred microns.

Supplementary Movie 8: Time-lapse of *A. fumigatus* CEA10 WT conidia treated with 2 µg/mL caspofungin plus 10 µg/mL 5,8- diHODE grown for 24 hours in liquid GMM at 37˚C. Images were collected every 15 minutes for 21 hours beginning at 3 hours post inoculation by phase microscopy with a 10X objective. Scale bars represent onehundred microns.
