# Supplementary material for: An oxylipin signal confers protection against antifungal echinocandins in pathogenic aspergilli
Source: Nat Commun. 2024 May 4;15:3770. doi: 10.1038/s41467-024-48231-2 (PMC11069582; doi:10.1038/s41467-024-48231-2)
Supplement: Supplementary file 13 — Reporting Summary [file 41467_2024_48231_MOESM13_ESM.pdf]

nature portfolio

Corresponding author(s): Nancy P. Keller

Last updated by author(s): Apr 6, 2024

Reporting Summary

Nature Portfolio wishes to improve the reproducibility of the work that we publish. This form provides structure for consistency and transparency in reporting. For further information on Nature Portfolio policies, see our Editorial Policies and the Editorial Policy Checklist.

Statistics

For all statistical analyses, confirm that the following items are present in the figure legend, table legend, main text, or Methods section.

n/a	Confirmed	
☐	☒	The exact sample size (n) for each experimental group/condition, given as a discrete number and unit of measurement
☐	☒	A statement on whether measurements were taken from distinct samples or whether the same sample was measured repeatedly
☐	☒	The statistical test(s) used AND whether they are one- or two-sided<br>*Only common tests should be described solely by name; describe more complex techniques in the Methods section.*
☒	☐	A description of all covariates tested
☐	☒	A description of any assumptions or corrections, such as tests of normality and adjustment for multiple comparisons
☐	☒	A full description of the statistical parameters including central tendency (e.g. means) or other basic estimates (e.g. regression coefficient) AND variation (e.g. standard deviation) or associated estimates of uncertainty (e.g. confidence intervals)
☐	☒	For null hypothesis testing, the test statistic (e.g. F, t, r) with confidence intervals, effect sizes, degrees of freedom and P value noted<br>*Give P values as exact values whenever suitable.*
☒	☐	For Bayesian analysis, information on the choice of priors and Markov chain Monte Carlo settings
☒	☐	For hierarchical and complex designs, identification of the appropriate level for tests and full reporting of outcomes
☒	☐	Estimates of effect sizes (e.g. Cohen's d, Pearson's r), indicating how they were calculated

Our web collection on statistics for biologists contains articles on many of the points above.

Software and code

Policy information about availability of computer code

Data collection	Micrographs were collected using NIS-Elements AR software (Version 5.30). Library preparation and RNA sequencing were performed by Novogene, Inc. using the TruSeq Stranded mRNA Library Prep Kit and Illumina Novaseq 6000 Platform. Adapter and low- quality reads were removed, and clean reads were mapped to the annotated genome of A. fumigatus Af293 obtained from FungiDB (release 52) using HiSAT2 (v2.0.5).
Data analysis	Hyphal branching and germling lysis assessments of phase micrographs were performed in NIS-Elements AR software (Version 5.30). Chitin quantification of CFW fluorescence micrographs was performed using ImageJ (Version 2.14.0/1.54f). For RNA-seq analysis, the read-count table was processed through DESeq2 (v1.20.0) to identify differentially regulated genes between mutant and wild type samples. Functional Catalogue (FunCat) analysis of differentially expressed genes was performed using FungiFun (v2.2.8) [https://elbe.hki-jena.de/fungifun/fungifun.php].

For manuscripts utilizing custom algorithms or software that are central to the research but not yet described in published literature, software must be made available to editors and reviewers. We strongly encourage code deposition in a community repository (e.g. GitHub). See the Nature Portfolio guidelines for submitting code & software for further information.

Data

Policy information about availability of data

All manuscripts must include a data availability statement. This statement should provide the following information, where applicable:

- Accession codes, unique identifiers, or web links for publicly available datasets
- A description of any restrictions on data availability
- For clinical datasets or third party data, please ensure that the statement adheres to our policy

All relevant meAll relevant methods of data collection and analysis are included in the methods section. The annotated reference genome of A. fumigatus Af293 was obtained from FungiDB (release 52) [https://fungidb.org/fungidb/app/]. The RNA sequencing data supporting the findings in this study have been deposited to the NCBI Gene Expression Omnibus with the identifier GSE231238 [https://www.ncbi.nlm.nih.gov/geo/query/acc.cgi?acc=GSE231238]. The list of differentially expressed genes from the RNA sequencing experiment is provided in Supplementary Data 1. All other data obtained to support the findings of this study are available within the article and supplementary materials or from the corresponding author upon request. Source data are provided with this paper.

Research involving human participants, their data, or biological material

Policy information about studies with human participants or human data. See also policy information about sex, gender (identity/presentation), and sexual orientation and race, ethnicity and racism.

Reporting on sex and gender	N/A
Reporting on race, ethnicity, or other socially relevant groupings	N/A
Population characteristics	N/A
Recruitment	N/A
Ethics oversight	N/A

Note that full information on the approval of the study protocol must also be provided in the manuscript.

Field-specific reporting

Please select the one below that is the best fit for your research. If you are not sure, read the appropriate sections before making your selection.

[x] Life sciences [ ] Behavioural & social sciences [ ] Ecological, evolutionary & environmental sciences

For a reference copy of the document with all sections, see nature.com/documents/nr-reporting-summary-flat.pdf

Life sciences study design

All studies must disclose on these points even when the disclosure is negative.

Sample size	Sample size in germling lysis experiments was determined by pilot experiments with WT A. fumigatus Af293 showing that significant differences in lysis between conditions could be detected using 3 replicates. Sample size for both hyphal branching and fluorescence quantification were selected based on published methods (Niu et al. Nat Commun. 2020 11:5158).
Data exclusions	No data was excluded.
Replication	Biological replication in all experiments was carried out as described in the methods and figure captions. Experimental replication was performed for all germling lysis experiments to ensure reproducibility of statistical findings.
Randomization	Treatment and control groups were inoculated into the same well-plates devices in a randomized manner to ensure no impact of sample location on experimental outcome.
Blinding	Data collection and analysis of germling lysis experiments and lateral branching experiments were performed by a blinded researcher. Data collection and analysis of oxylipin quantification was performed by a researcher blinded to treatment.

Reporting for specific materials, systems and methods

We require information from authors about some types of materials, experimental systems and methods used in many studies. Here, indicate whether each material, system or method listed is relevant to your study. If you are not sure if a list item applies to your research, read the appropriate section before selecting a response.

Materials & experimental systems

n/a	Involved in the study
☒	☐ Antibodies
☒	☐ Eukaryotic cell lines
☒	☐ Palaeontology and archaeology
☒	☐ Animals and other organisms
☒	☐ Clinical data
☒	☐ Dual use research of concern
☒	☐ Plants

Methods

n/a	Involved in the study
☒	☐ ChIP-seq
☒	☐ Flow cytometry
☒	☐ MRI-based neuroimaging

Plants

Seed stocks	*Report on the source of all seed stocks or other plant material used. If applicable, state the seed stock centre and catalogue number. If plant specimens were collected from the field, describe the collection location, date and sampling procedures.*
Novel plant genotypes	*Describe the methods by which all novel plant genotypes were produced. This includes those generated by transgenic approaches, gene editing, chemical/radiation-based mutagenesis and hybridization. For transgenic lines, describe the transformation method, the number of independent lines analyzed and the generation upon which experiments were performed. For gene-edited lines, describe the editor used, the endogenous sequence targeted for editing, the targeting guide RNA sequence (if applicable) and how the editor was applied.*
Authentication	*Describe any authentication procedures for each seed stock used or novel genotype generated. Describe any experiments used to assess the effect of a mutation and, where applicable, how potential secondary effects (e.g. second site T-DNA insertions, mosiacism, off-target gene editing) were examined.*
